# The Impact of Genes and Environment on Brain Ageing in Males Aged 51 to 72 Years

**DOI:** 10.3389/fnagi.2022.831002

**Published:** 2022-04-15

**Authors:** Nathan A. Gillespie, Sean N. Hatton, Donald J. Hagler, Anders M. Dale, Jeremy A. Elman, Linda K. McEvoy, Lisa T. Eyler, Christine Fennema-Notestine, Mark W. Logue, Ruth E. McKenzie, Olivia K. Puckett, Xin M. Tu, Nathan Whitsel, Hong Xian, Chandra A. Reynolds, Matthew S. Panizzon, Michael J. Lyons, Michael C. Neale, William S. Kremen, Carol Franz

**Affiliations:** ^1^Virginia Institute for Psychiatric and Behaviour Genetics, Department of Psychiatry, Virginia Commonwealth University, Richmond, VA, United States; ^2^QIMR Berghofer Medical Research Institute, Brisbane, QLD, Australia; ^3^Department of Psychiatry, University of California, San Diego, La Jolla, CA, United States; ^4^Center for Behavior Genetics of Aging, University of California, San Diego, La Jolla, CA, United States; ^5^Department of Neurosciences, University of California, San Diego, La Jolla, CA, United States; ^6^Department of Radiology, University of California, San Diego, La Jolla, CA, United States; ^7^Center for Multimodal Imaging and Genetics, University of California, San Diego, La Jolla, CA, United States; ^8^Halıcıoğlu Data Science Institute, University of California, San Diego, La Jolla, CA, United States; ^9^Herbert Wertheim School of Public Health and Human Longevity Science, University of California, San Diego, La Jolla, CA, United States; ^10^Mental Illness Research Education and Clinical Center, VA San Diego Healthcare System, San Diego, CA, United States; ^11^National Center for PTSD, VA Boston Healthcare System, Boston, MA, United States; ^12^Department of Psychiatry and Biomedical Genetics Section, Boston University School of Medicine, Boston, MA, United States; ^13^Department of Biostatistics, Boston University School of Public Health, Boston, MA, United States; ^14^Department of Psychology, Boston University, Boston, MA, United States; ^15^School of Education and Social Policy, Merrimack College, North Andover, MA, United States; ^16^Division of Biostatistics and Bioinformatics, Herbert Wertheim School of Public Health and Human Longevity Science, University of California, San Diego, La Jolla, CA, United States; ^17^Department of Epidemiology and Biostatistics, Saint. Louis University, St. Louis, MO, United States; ^18^Research Service, VA St. Louis Healthcare System, St. Louis, MO, United States; ^19^Department of Psychology, University of California, Riverside, Riverside, CA, United States; ^20^Department of Psychological and Brain Sciences, Boston University, Boston, MA, United States; ^21^Department of Biological Psychology, Free University of Amsterdam, Amsterdam, Netherlands; ^22^Center of Excellence for Stress and Mental Health, VA San Diego Healthcare System, La Jolla, CA, United States

**Keywords:** predicted brain ageing, twin, gene, longitudinal predicted brain aging, MRI, development, cognitive decline, Alzheimers’s disease

## Abstract

Magnetic resonance imaging data are being used in statistical models to predicted brain ageing (PBA) and as biomarkers for neurodegenerative diseases such as Alzheimer’s Disease. Despite their increasing application, the genetic and environmental etiology of global PBA indices is unknown. Likewise, the degree to which genetic influences in PBA are longitudinally stable and how PBA changes over time are also unknown. We analyzed data from 734 men from the Vietnam Era Twin Study of Aging with repeated MRI assessments between the ages 51–72 years. Biometrical genetic analyses “twin models” revealed significant and highly correlated estimates of additive genetic heritability ranging from 59 to 75%. Multivariate longitudinal modeling revealed that covariation between PBA at different timepoints could be explained by a single latent factor with 73% heritability. Our results suggest that genetic influences on PBA are detectable in midlife or earlier, are longitudinally very stable, and are largely explained by common genetic influences.

## Introduction

Brain magnetic resonance imaging (MRI) data are increasingly used to predict brain ageing. In turn, predicted brain ageing (PBA) is being used to estimate lifespan, to characterize accelerated ageing, and to identify individuals with mild cognitive impairment and the likelihood of progression to dementia including Alzheimer’s Disease (Deary et al., 2009; Salthouse, 2010; Vos et al., 2012; Gaser et al., 2013; Fjell et al., 2014; Lowe et al., 2016; Liem et al., 2017; Cole et al., 2019; Elliott et al., 2019; de Lange and Cole, 2020). This approach works by relying on machine learning to estimate associations between imaging data and chronological age in training samples of varying ages (Cole and Franke, 2017). Using supervised learning algorithms, these associations are then applied to estimate PBA or predicted brain age difference (PBAD) (the difference between predicted and chronological age) in independent samples. Not only does the approach assume that MRI of neuroanatomical degeneration reflects poorer brain health and risk of neurodegenerative diseases (McEvoy et al., 2009; Cole et al., 2019; Wang et al., 2019) but that individual differences in brain aging stem from biological processes influencing lifespan and age-related diseases explained by genetic and environmental influences (Cole et al., 2019). However, very little is known about the relative contribution of genetic and environmental influences on PBA or PBAD and how these may change over time.

We are aware of only two twin reports examining the heritability of PBA and PBAD; Cole’s (Cole et al., 2017) cross-sectional analysis of 62 female twins at mean age 62 years, and Brouwer’s (Brouwer et al., 2021) longitudinal analysis of 673 twins aged 10–23 years. The latter reported PBAD heritabilities up to 79% as well as longitudinal genetic correlations based on gray matter density and cortical thickness ranging 0.46–0.68. In addition to demonstrating heritability, these results suggest a combination of stable and age-varying genetic influences on brain aging at least in adolescents and young adults. Apart from Brouwer’s analysis of adolescent and young adult twin data, to our knowledge, there have been no twin reports that have (i) estimated the genetic and environmental influences on PBA and PBAD on older populations, or (ii) tested developmental hypotheses regarding the stability of genetic influences on brain ageing. Given the emphasis on early detection of neurodegenerative diseases (Daviglus et al., 2010; Albert et al., 2011; Golde et al., 2011; Sperling et al., 2011a,b), we sought to address these gaps in our understanding.

Following the reports of Cole et al. (2017) and Brouwer et al. (2021) and based on published heritability estimates for cortical and subcortical volume (Baare et al., 2001; Wright et al., 2002; Peper et al., 2007; Kremen et al., 2010; Brouwer et al., 2014; Renteria et al., 2014; Satizabal et al., 2019), cortical thickness (Thompson et al., 2001; Kremen et al., 2010; Kremen et al., 2013a; Vuoksimaa et al., 2015), cortical surface area (Kremen et al., 2010; Eyler et al., 2011; Kremen et al., 2013a; Brouwer et al., 2014; Vuoksimaa et al., 2015), and diffusion MRI metrics (Elman et al., 2017; Gillespie et al., 2017; Hatton et al., 2018b), we hypothesized that MRI-based whole-brain indicators of PBA and PBAD should be heritable. Next, we tested developmental hypotheses. Theories of somatic mutation predict an accumulation of unrepaired cellular and molecular damage arising from genome instability during a single generation (Kirkwood, 1977; Morley, 1998; Kirkwood, 2005), which is consistent with autoregression (Guttman, 1954; Eaves et al., 1986; Boomsma and Molenaar, 1987; Boomsma et al., 1989). If changes in brain ageing do indeed stem from the accumulation of age-related genetic and environmental influences, the task is to determine how well autoregression explains observed PBA data. Alternatively, it is plausible that genetic and environmental influences in PBA are time-invariant and better explained by common or independent pathway theories (Neale and Cardon, 1992).

Our aim, therefore, was to explore the etiology of PBA (and PBAD) in a sample of middle- to later-age men with longitudinal MRI assessments. In addition to estimating PBA heritability, we tested competing hypotheses to explain best the longitudinal changes in genetic and environmental influences.

## Materials and Methods

### Subjects

Participants comprise middle-aged male twins who underwent MRI scanning as part of the Vietnam Era Twin Study of Aging (VETSA) (Kremen et al., 2013b). Wave 1 took place between 2001 and 2007 (Kremen et al., 2006; mean age = 56.1, SD = 2.6, range = 51.1–60.2). Wave 2 occurred approximately 5.5 years later (mean age = 61.8, SD = 2.6, range = 56.0–65.9). Wave 3 occurred approximately 5.7 years later (mean age = 67.5, SD = 2.6, range = 61.4–71.7). All participants were concordant for US military service at some time between 1965 and 1975. Nearly 80% reported no combat experience. The sample is 88.3% non-Hispanic white, 5.3% African-American, 3.4% Hispanic, and 3.0% “other” participants. Based on data from the US National Center for Health Statistics, the sample is very similar to American men in their age range with respect to health and lifestyle characteristics (Schoeneborn and Heyman, 2009).

### Ethics

Written informed consent was obtained from all participants. The University of California, San Diego, Human Research Protection Program Institutional Review Board approved the proposal to collect these data (Project #150572, 150537, 140361, 071446, 031639, and 151333). Data are publicly available through requests at the VETSA website.^1^

### MRI Acquisition

A description of the MRI acquisition and derivation of the predicted brain age (PBA) and predicted brain age difference (PBAD) endophenotypes are provided in the Supplement. Discussed in detail elsewhere (Hatton et al., 2018a), PBA was estimated using the Brain-Age Regression Analysis and Computation Utility software BARACUS v0.9.4 (Github Inc, 2017; Liem et al., 2017). PBAD scores were calculated by subtracting PBA, also referred to as “stacked-anatomy” brain age in BARACUS, from the chronological age. A negative PBAD is indicative of brain age estimated to be older than one’s chronological age. Briefly, this approach works by relying on machine learning to estimate associations between imaging data and chronological age in training samples of varying age. We used the BIDS-mode docker on Ubuntu 16.04 using the default database that was trained on N = 1,166 subjects with no objective cognitive impairment (566 women/600 men, mean age 59.1 years, SD 15.2, range 20–80 years; Hatton et al., 2018a).

We note that while supervised machine learning algorithms such as BARACUS can detect informative multivariate patterns, the relative contributions of individual regions are not tested. Therefore, no inferences are made regarding particular regions of interest that might be responsible for individual differences in PBA or PBAD.

As noted in section “Subjects” there was considerable variation in chronological age at each wave and overlap in age ranges between the three assessments. Given the variation and overlap, longitudinal analysis of these wave-based data would therefore preclude any meaningful understanding of age-related changes. Ignoring irregular spacing between time intervals in longitudinal modeling can lead to biased parameter estimates (Estrada and Ferrer, 2019). Rather than employing definition variables to account for individual differences in age at assessment and irregular timer intervals (Mehta and Neale, 2005), our solution was to recode each subject’s score according to their chronological age at assessment. Thus, for example, if two subjects “a” and “b” were both aged 60 at VETSA 1 and 2, respectively, each would be assigned a PBA score for age 60. Since each subject contributed a maximum of three data points between ages 51 and 72, this creates missing data for which Full Information Maximum Likelihood is well suited to handling. However, to reduce sparse data while maintaining computational efficiency, our “age-anchored” PBA and PBAD scores were re-coded to one of four age intervals according to each individual’s age at assessment: 51–55; 56–60; 61–65; and 66–72 years.

There were 260, 251, and 126 subjects with PBA scores at one, two and three age intervals, respectively. Since there were only 3 VETSA assessments, no subjects had data from all four age intervals. Five participants were ascertained twice in the same 5-year age interval. Only their first observation was included. Prior to twin modeling all PBA and PBAD scores were residualized for the location and scanner differences (i.e., 1.5T vs 3T), age at assessment and ethnicity using the umx_residualize function in the umx software package (Bates et al., 2019), and given the range in birth year (1943–1955), residuals were also adjusted for cohort effects.

### Statistical Analyses

The OpenMx_2.9.9.1_ software package (Boker et al., 2011) in R_3.4.1_ (R Development Core Team, 2018) was used to estimate correlations between the PBA scores and to fit univariate and multivariate genetic twin models (Neale and Cardon, 1992). The OpenMx code used for the multivariate analyses is included in the Supplement. Given the numbers of incomplete twin pairs (see Supplementary Table 1), methods such as Weighted Least Squares would result in significant listwise deletion thereby altering the accuracy of the PBA and PBAD means and variances. Fortunately, the raw data Full Information Maximum Likelihood (FIML) option in OpenMx_2.9.9.1_ (Boker et al., 2011) has the advantage of not only being robust to violations of non-normality but also enables analysis of missing or incomplete data as well as the direct estimation of covariate effects. More accurate means and variance improve the estimation of the variances and covariance structure used to test our competing hypotheses.

### Univariate Analyses

**FIGURE 1:**
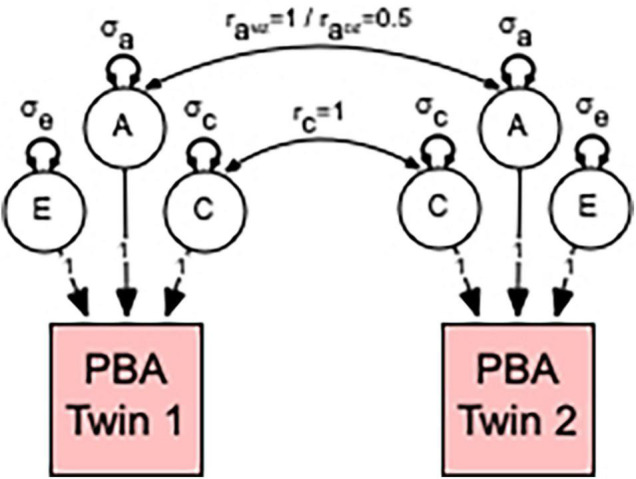
Univariate model to estimate the relative contribution of genetic and environmental influences in predicted brain ageing (PBA). Individual differences in PBA are decomposed into three sources of variation: additive genetic (A); common or shared environmental influences (C); and unshared or random environmental influences as well as measurement error (E). This decomposition is achieved by specifying the expected genetic and environmental correlations between monozygotic (MZ) and dizygotic (DZ) twin pairs. MZ twin pairs are genetically identical, whereas DZ twin pairs share, on average, half of their genes. Therefore, the MZ and DZ twin pair correlations (r_aMZ_ and r_aDZ_) for additive genetic effects are fixed to 1.0 and 0.5, respectively. This model also assumes that shared environmental effects are equally correlated (r_c_ = 1) in MZ and DZ twin pairs. Non-shared environmental influences are by definition uncorrelated within twin pairs (r_e_ = 0). Note that our method of estimating the relative contribution of genetic and environmental influences in PBA proceeds by estimating the additive genetic (σ_a_), shared environmental (σ_c_), and non-shared environmental (σ_e_) variances for the A, C, and E latent factors. The size or contribution of these σ_e_, σ_c_, and σ_e_ variance components to the phenotype are assumed to be equal within twin pairs.

In univariate analyses, the total variation in each PBA score was decomposed into additive (A) heritability, shared or common environmental (C), and non-shared or unique (E) environmental variance components (see Figure 1). This approach is referred to as the “ACE” variance component model. The decomposition is achieved by exploiting the expected genetic and environmental correlations between MZ and DZ twin pairs; MZ twin pairs are genetically identical, whereas DZ twin pairs share, on average, half of their genes. Therefore, MZ and DZ twin pair correlations (r_*A*_) for additive genetic effects are fixed to 1.0 and 0.5, respectively. The modeling assumes that shared environmental effects (C) are equal in MZ and DZ twin pairs, whereas non-shared environmental effects (E) are by definition uncorrelated and include measurement error.

### Multivariate Analyses to Test Competing Theories

This univariate method is easily extended to the multivariate case to estimate the size and significance of genetic and environmental influences within and between PBA over time.

**FIGURE 2:**
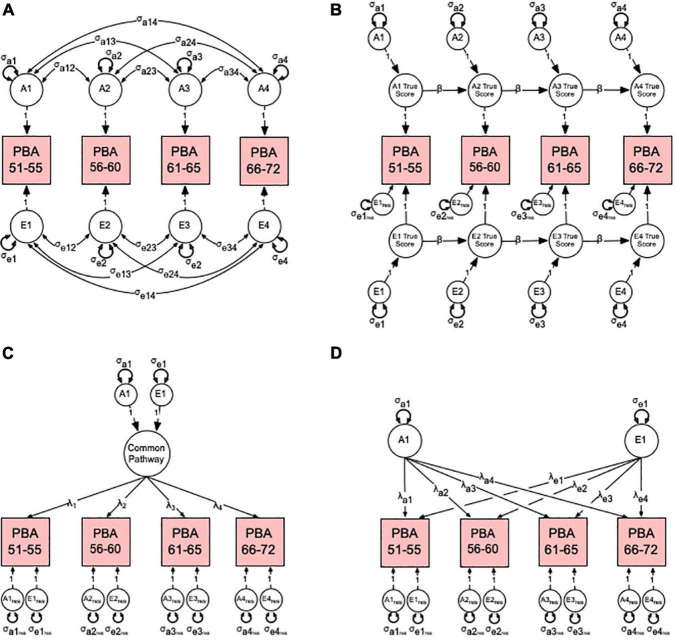
Multivariate correlated factors **(A)** and competing hypothetical models to explain the sources of variance-covariance between the predicted brain age (PBA) scores. Competing models include **(B)** the auto-regression, **(C)** common pathway, and **(D)** independent pathway models. For brevity, only latent additive genetic (A1–4) and non-shared environmental (E1–4) factors are shown. **(A)** The multivariate correlated factor model estimates the size of the latent genetic and environmental variances and covariances (double-headed arrows). It is atheoretical and makes no prediction about the nature of change in PBA over time. **(B)** In the autoregression model, the time-specific genetic (σ_a1–4_) and environmental (σ_e1–4_) variance components or “innovations” for each genetic (A1–4) and environmental (E1–4) latent factor true score are estimated along with each variable’s residual or error variance (σ_*e*1res–e4res_). Also estimated are the autoregression or causal coefficients (β) from one latent true score to the next. **(C)** In the common pathway model, the genetic (σ_a1_) and environmental (σ_e1_) variance components for the common pathway, the factor loadings (λ_1–4_), and latent genetic and environmental residuals (σ_a1res–a4res_, σ_e1res–e4res_) are estimated. **(D)** Finally, in the independent pathway model, genetic (σ_a1_) and environmental (σ_e1_) variance components are estimated independently with their factor loadings (λ_a1–4_, λ_e1–4_), and latent genetic and environmental residuals (σ_*a*1res–a4res_, σ_e1res–e4res_). See Supplement for more detailed modeling description.

In order to have a reference for contrasting and choosing the best fitting theoretical model, we first fitted a multivariate ACE “correlated factors” (Figure 2A) before fitted competing autoregression (Figure 2B), common (Figure 2C) and independent pathway (Figure 2D) models See Supplement for detailed modeling explanation. Given that (i) the machine learning method used here to calculate PBA and PBAD relied on a cognitively normal training sample and (ii) our twin analyses relied on a community-dwelling (non-clinically) ascertained sample, we therefore, refer to all A, C, and E variance components as genetic and environmental “influences”, which assumes any observed variation in normal brain ageing comprises both risk and protective factors.

### Model Fit

The best-fitting model was determined using a using a likelihood ratio test and the Akaike’s Information Criterion (AIC) (27). For each best-fitting univariate and multivariate model, the parameters were then successively fixed to zero and their significance determined using a likelihood ratio chi-square test.

## Results

The numbers of complete and incomplete twin pairs by zygosity are shown in Supplementary Table 1. Descriptive statistics for each PBA score before and after residualization of the means and variances are shown in Supplementary Table 2.

### Strength of Association

All phenotypic correlations between the PBA scores at each age interval were high and ranged from 0.67 to 0.76 (see Table 1).

**TABLE 1:** Predicted brain age (PBA) phenotypic polyserial correlations.

	(1)	(2)	(3)	(4)
(1) PBA 51–55	1			
(2) PBA 56–60	0.67	1		
(3) PBA 61–65	0.76	0.74	1	
(4) PBA 66–72	0.67	0.72	0.75	1

*Polyserial correlations represent the associations between the underlying liability rather than observed phenotypic distributions (Pearson, 1900; Pearson and Pearson, 1922).*

### Twin Pair Correlations

Table 2 shows the twin pair correlations by zygosity for the PBA scores at each age interval. If familial aggregation was entirely attributable to shared family environments, then monozygotic (MZ) and dizygotic (DZ) twin pair correlations would be statistically equal. In contrast, if familial aggregation was entirely attributable to shared additive (or non-additive) genetic factors, then DZ correlations would be 1/2 (or less) the size of the MZ twin pair correlations. Here, DZ twin pair correlations ranged from 0.1 to 0.6 and were ∼1/3 the size of the MZ twin pair correlations.

**TABLE 2:** Predicted brain age monozygotic and dizygotic twin pair polyserial correlations (corrMZ and CorrDZ) along with standardized variance components and 95% confidence intervals components for the best-fitting additive genetic (A) and non-shared environment (E) univariate models.

	corr_MZ_	(95% CIs)	Corr_DZ_	(95% CIs)	A	(95% CIs)	E	(95% CIs)
PBA 51–55	0.68	(0.53–0.78)	0.13	(−0.19 to 0.42)	0.64	(0.54–0.69)	0.36	(0.31–0.46)
PBA 56–60	0.70	(0.59–0.78)	0.29	(0.11–0.46)	0.71	(0.61–0.79)	0.29	(0.21–0.39)
PBA 61–65	0.60	(0.49–0.70)	0.20	(0.01–0.38)	0.58	(0.45–0.68)	0.42	(0.32–0.55)
PBA 66–72	0.66	(0.55–0.75)	0.18	(–0.07 to 0.58)	0.61	(0.47–0.71)	0.39	(0.29–0.53)

### Univariate Analyses

Predicted brain ageing univariate model fitting results are shown Supplementary Table 3. At each age interval, the “AE” model with no common environmental effects provided the best fit. Familial aggregation in each PBA score could be entirely explained by additive genetic influences (A) ranging from 59 to 75% (see Table 2). All remaining variation was explained by non-shared environmental influences.

### Multivariate Analyses

Both the autoregression and independent pathway models fitted the data poorly as judged by the significant change in their likelihood chi-squared ratios (see Supplementary Table 4). In contrast, the changes in the likelihood for the 1- and 2-factor common pathway models were not significant. Here, the 1-factor common pathway model provided a better comparative fit as judged by the lower AIC, and in subsequent modeling (see Supplementary Table 5), both the “CE” and “E” sub-models deteriorated significantly whereas the “AE” model yielded a non-significant likelihood ratio chi-square difference as well as the lowest AIC.

**FIGURE 3:**
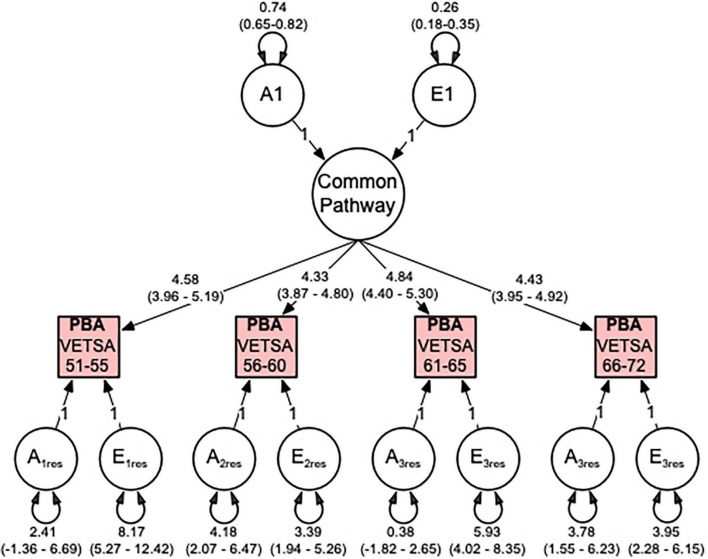
Predicted brain age (PBA) best fitting common pathway (CP) multivariate model comprising additive genetic (A) and non-shared environment (E) variance components. Illustrated are the genetic and environmental variance components for the common pathway, the factor loadings from the CP to the observed PBA phenotypes, and the genetic and environmental residual variance components. All variance components are standardized and include 95% confidence intervals.

Thus, our multivariate analyses indicate that correlations between the PBA measures across time are best explained by a single factor, which can be explained 74% additive genetic and 26% non-shared environmental influences (see Figure 3). Total genetic variances (common and residual influences) in PBA at ages 51–55, 56–60, 61–65, and 66–72 were estimated to be 57, 69, 60, and 67%, respectively. For PBA at ages 61–65, the residual genetic variance was non-significant, indicating that genetic variance here is entirely captured by the common factor.

Genetic correlations between the four PBA scores were high and ranged from 0.78 to 0.92 (Table 3) indicating that the same genes are largely influencing PBA across time. In contrast, the environmental correlations were moderate to high, ranging from 0.45 to 0.58 (Table 3) suggesting that large proportions of the environmental influences are unique to each age interval.

**TABLE 3:** Predicted brain age additive genetic (below diagonal) and non-shared environmental correlations based on the best fitting “AE” 1-factor common pathway model.

	(1)	(2)	(3)	(4)
(1) PBA 51–55	1	0.48	0.45	0.47
(2) PBA 56–60	0.82	1	0.54	0.58
(3) PBA 61–65	0.92	0.87	1	0.53
(4) PBA 66–72	0.83	0.78	0.88	1

We then applied the same univariate and multivariate modeling pipeline to the PBAD scores. All results are shown in the Supplementary Tables 6–10. Not only were the patterns of additive genetic and non-shared environmental factor correlations in the best fitting 1-factor common pathway “AE” model for PBAD nearly identical to PBA, the heritability of the common pathway was identical at 74%.

## Discussion

Individual differences in MRI-based estimates of PBA and PBAD are highly heritable, with genetic influences accounting for approximately three-quarters of the overall variance. The genetics of PBA are also highly correlated across time and these correlations can be best explained by a common set of heritable influences. Consequently, efforts to identify common molecular variants in PBA (Smith et al., 2020) may not require age-stratified samples. Our findings are also consistent with the hypothesis that common genetic influences explain most of the individual differences in brain ageing beginning in midlife and onward.

We also found that PBA could not be explained by shared environmental influences that drive twin pair similarity. Twins reared together are ideal controls for environmental influences that were shared during infancy and youth, continue to be shared, or continue to exert an impact. Naturally, as twins age and spend less time together, one would expect the number of directly shared environmental influences, relative to the time in their lives when they were reared together, to be diminished. Thus, in terms of individual differences in brain ageing, environments shared between family members that increase twin pair similarity, e.g., household and early rearing environments, parental income and SES (van der Loos et al., 2013; Davies et al., 2015), lack enduring or persistent effects and are of less importance than environments that are unique to individuals, e.g., diet, drug use or allostatic stressors such as negative life events (Hatton et al., 2018a). Indeed, we have previously shown that having more favorable and modifiable lifestyle behaviors such as a good diet, physical activity, social engagement, and less nicotine and alcohol consumption predict less advanced brain age and less AD-like brain aging (Franz et al., 2021; Whitsel et al., 2021). These findings may have implications concerning the efficacy of community-based versus individually targeted efforts to slow rates of brain ageing.

The hypothesis regarding accumulative environmental and molecular influences predicted by somatic mutation theories that ought to be captured by autoregression modeling was not supported. Instead, our data were consistent with what is perhaps a counterintuitive explanation. To the extent that any unrepaired damage is linked to genetic variation in our global indices of PBA, our modeling provided little support for autoregression features or accumulation of age-related or age-specific genetic influences over time. Likewise, we found no evidence to support the hypothesis that age-specific environmental influences are accumulative.

Instead, our best-fitting model suggests that brain ageing is best explained by stable genetic and environmental influences acting *via* a highly heritable common pathway accounting for most of the individual differences over a 21-year period. Our modeling makes no prediction regarding the number of genes likely involved in brain ageing. However, given recent genome wide association scan (GWAS) findings based on multiple brain ageing indices (Smith et al., 2020), including a GWAS of lifespan (Timmers et al., 2019), we speculate that ageing processes are highly polygenic. Our statistically derived common pathway should not be interpreted to represent any identifiable biological structure(s) governing this supervised learning index of ageing. It is, instead, consistent with Kirkwood’s theory of a centrally regulated process of ageing, which under selection, has evolved to optimize the “allocation of metabolic resources across core processes like growth, reproduction, and maintenance” (Kirkwood, 2005). Kirkwood also argued that “network” theories of ageing used to describe multiple processes (Kirkwood, 1977, 2005) ought to distinguish upstream mechanisms that set ageing in motion from downstream mechanisms that affect ageing at the cellular level toward the end of life (Kowald and Kirkwood, 1996). The high genetic correlation of r_*g*_ = 0.72 between ages 51–55 and 66–72 suggests, broadly, that genetic influences underpinning any putative “upstream” and “downstream” processes are mostly shared in common.

We have demonstrated that having more negative life events, particularly relating to interpersonal relationships, is associated with advanced PBA, i.e., higher predicted brain age relative to chronological age (Hatton et al., 2018a).

### Limitations

Our results should be interpreted in the context of four potential limitations.

First, our hypothesis testing was not exhaustive. If PBA is related to rates of cellular or molecular ageing (Kirkwood, 2005), plausibly, genetic and environmental influences could unfold over time, and be better explained by growth processes (Nesselroade and Baltes, 1974; McArdle, 1986; McArdle and Epstein, 1987; Duncan and Duncan, 1991; Duncan et al., 1994). Although each twin pair was assessed on the same scanner on each measurement occasion, MRI data were collected on different scanners (i.e., 1.5T at VETSA 1 vs 3T at VETSA 2 and 3) resulting in likely measurement non-invariance across assessments. Consequently, data were residualized for these and other covariate effects. This resulted in the loss of interpretable mean and variance information necessary for latent growth curve modeling.

Second, our data were limited to midlife and early old age. Therefore, the stability in the genetic and environmental influences observed between ages 51 and 72 years may not generalize to other periods in the life course. For example, it is conceivable that genetic and environmental autoregression processes may have occurred before our first assessment (Elliott et al., 2019). There may also exist sub-groups of individuals for whom different autoregressions or hybrid auto-regression plus common factor models provide a better explanation of change over time. These hypotheses can only be resolved with additional data, e.g., data collected earlier in life, and are not within the scope of the current data.

Third, of the age at interview distribution at each of the VETSA waves spanned a decade. As mentioned in the “Materials and Methods”, rather than employing definition variables to account for individual differences in age at assessment, our solution was to recode each subject’s PBA and PBAD scores according to their chronological age at assessment. Our results should therefore, be interpreted as the average change of individuals with the 4-year age intervals. We did, however, repeat our analyses using the wave-based data whereby the assessment occasion was treated as a different time point (i.e., the VETSA interviews at waves 1, 2, and 3) while modeling age at assessment as a covariate. Here again, we found that the common pathway provided the best fit to the data.

Finally, our results may not generalize to women or ethnic minorities. We know of no other genetically informative twin studies with comparable and longitudinal MRI data. The uniqueness and size of our sample is a key strength of the VETSA cohort.

## Conclusion

This is the first study to explore the genetic and environmental influences on PBA in a longitudinal sample. We assessed males age 51–72 years and report three major findings. First, measures of PBA were highly correlated across time. Second, the heritability estimates based on univariate twin analyses ranged from 59 to 74%. Finally, there was no evidence that PBA could be explained by an accumulation of age-specific genetic or environmental influences. Instead, genetic influences at each age interval were highly correlated and captured by a single, common factor with a heritability of 73%. Future analyses should explore the sources of genetic and environmental covariation between brain ageing and other complex behaviors related to cognitive decline.

## Data Availability Statement

OpenMx software coding used for the multivariate analyses is included in the Supplementary Material. All additional OpenMx software code is available upon request. Data are publicly available through requests at the VETSA website (http://www.vetsatwins.org).

## Ethics Statement

The University of California, San Diego, Human Research Protection Program Institutional Review Board approved the proposal to collect these data (Project #150572, 150537, 140361, 071446, 031639, and 151333). The patients/participants provided their written informed consent to participate in this study.

## Author Contributions

NAG, JAE, MSP, MJL, MCN, WSK, and CF were responsible for generating the hypotheses. SNH, DJH, AMD, JAE, CF-N, NW, MJL, WSK, and CF were responsible for data collection. NAG and MCN were responsible for the statistical analyses. NAG, SNH, DJH, AMD, JAE, LKM, LTE, CF-N, MWL, REM, OKP, XMT, HX, CAR, MSP, MJL, MCN, WSK, and CF were responsible for manuscript editing. All authors listed have made a substantial, direct, and intellectual contribution to the work, and approved it for publication.

## Conflict of Interest

NAG hold equity in Cassava Sciences, Inc. LKM holds equity in CorTechs Labs, Inc. The remaining authors declare that the research was conducted in the absence of any commercial or financial relationships that could be construed as a potential conflict of interest.

## Publisher’s Note

All claims expressed in this article are solely those of the authors and do not necessarily represent those of their affiliated organizations, or those of the publisher, the editors and the reviewers. Any product that may be evaluated in this article, or claim that may be made by its manufacturer, is not guaranteed or endorsed by the publisher.

## Footnotes

1
http://www.vetsatwins.org
